# Effects of deficit irrigation and biostimulants on melon productivity and quality in semi‐arid conditions

**DOI:** 10.1002/jsfa.70674

**Published:** 2026-04-21

**Authors:** Jefferson dos Santos Gomes Calaça, Jean Carlos Nogueira, José Raliuson Inácio Silva, Denizard Oresca, Alan Cezar Bezerra, Raquele Mendes de Lira, George do Nascimento Araújo Júnior, Luiz Guilherme Medeiros Pessoa, Agda Raiany Mota dos Santos, Sidney Antônio da Silva, Guilherme Heverton Lima e Silva, Douglas Nogueira Lima, Eduardo Soares de Souza

**Affiliations:** ^1^ Academic Unit of Serra Talhada Federal Rural University of Pernambuco Serra Talhada Pernambuco Brazil; ^2^ Centre of Agrarian Science Federal University of Alagoas Rio Largo Alagoas Brazil; ^3^ Federal Rural University of Pernambuco Recife Pernambuco Brazil

**Keywords:** abiotic stress mitigation, irrigation efficiency, saline water, crop resilience, sustainable intensification, semi‐arid farming systems

## Abstract

**BACKGROUND:**

Water scarcity and soil salinization are major constraints to irrigated agriculture in semi‐arid regions, affecting crop productivity and fruit quality. Combining deficit irrigation with biostimulant application has emerged as a promising strategy to improve water use efficiency and plant stress tolerance. This study evaluated the effects of irrigation levels and amino acid‐based biostimulant rates on physiological responses, yield, and postharvest quality of melon (*Cucumis melo* L.) grown under semi‐arid conditions.

**RESULTS:**

The experiment was conducted during the rainy, transition, and dry seasons of 2023 using a randomized block design with three irrigation levels (50%, 75%, and 100% of soil available water) and four biostimulant rates (0, 4, 6, and 8 L ha^−1^). Stomatal conductance (gs) and leaf temperature (LT) were monitored during the crop cycle, while yield and fruit quality attributes were evaluated at harvest. Irrigation at 75% of available water resulted in yields comparable to those obtained with full irrigation, exceeding 34 Mg ha^−1^ while reducing water input. The application of 4 L ha^−1^ of biostimulant increased melon productivity by up to 32.58% compared with the control treatment. Increasing biostimulant rates promoted reductions in gs, suggesting improved stomatal regulation under stress conditions, while LT remained stable across treatments. Fruit quality parameters were maintained even under saline conditions and deficit irrigation.

**CONCLUSION:**

Moderate deficit irrigation combined with optimized biostimulant application improves melon tolerance to water and salt stress without compromising fruit quality. This strategy enhances water use efficiency and represents a sustainable alternative for melon production in semi‐arid environments. © 2026 The Author(s). *Journal of the Science of Food and Agriculture* published by John Wiley & Sons Ltd on behalf of Society of Chemical Industry.

## INTRODUCTION

Climate change has intensified the frequency and severity of extreme events, such as prolonged droughts and irregular rainfall, representing one of the greatest current challenges for irrigated agriculture on a global scale.^1^ The increase in average air temperature, combined with the reduced predictability of rainfall patterns, has caused significant water imbalances, especially in arid and semi‐arid regions, where agricultural production already depends heavily on irrigation to remain viable.^2^ In these regions, water scarcity is aggravated by high evaporative demand, making efficient water resource management a determining factor for the sustainability of production systems.^3^ In Brazil, this scenario is particularly evident in the semi‐arid northeast, where agriculture faces limitations imposed both by low water availability and by the progressive salinization of soils and irrigation water.^4^ Under these conditions, the adoption of management strategies that reconcile agricultural productivity and rational water use becomes essential to mitigate climate impacts and ensure the resilience of agricultural systems, especially in crops of high economic value, such as melons (*Cucumis melo* L.).

In the global context, melon cultivation stands out as one of the most economically important crops among the species of the Cucurbitaceae family, ranking fourth among the most traded fruits on the world market.^5^ In Brazil, production is concentrated predominantly in the northeast region, where the states of Rio Grande do Norte, Ceará, Bahia, and Pernambuco account for more than 93% of national production, due to favorable soil and climate conditions and the adoption of intensive irrigation systems.^6^ Among the main types sold are melons from the botanical group inodorus, such as the Amarelo, Honey Dew, and Pele de Sapo cultivars, characterized by a longer postharvest life, followed by aromatic melons from the Cantaloupe, Galia, and Charentais groups, widely valued for the sensory quality of the fruit.^5^

Globally, approximately 70–75% of available fresh water is used for irrigated agriculture, which makes careful management of irrigation depths applied to agricultural crops essential.^7^ Both excess and deficiency of water can compromise plant development, resulting in physiological and productive losses.^8^ In regions characterized by limited water availability, such as arid and semi‐arid areas, it is necessary to adopt management strategies that reduce water waste and simultaneously maximize production efficiency.^7^ Studies conducted in semi‐arid environments highlight the sensitivity of melon plants to water stress at critical phenological stages. Wang *et al*.,^9^ evaluating melon cultivation in China, demonstrated that maintaining soil water content above 55% of field capacity during the flowering and fruiting stages, and not less than 65% during fruit filling, is essential to avoid significant yield reductions. Similarly, Yavuz *et al*.^10^ emphasized that in hot climates, continuous irrigation during the growing season is essential to preserve melon productivity.

Given these limitations imposed by water scarcity, management practices that exploit plants' adaptive capacity to water stress have gained prominence in the scientific literature. In this context, deficit irrigation emerges as a strategy based on the controlled application of water below the total demand of the crop, assuming that moderate water deficits can induce physiological adjustments, such as reduced stomatal conductance and increased water use efficiency, without necessarily causing significant losses in productivity.^8^ Thus, understanding the physiological and productive responses of crops under different deficit irrigation regimes is fundamental for the development of more efficient and sustainable water management strategies, especially in environments with limited water resources.^11^

In addition to irrigation management, biostimulation has also been used as an alternative to help plants cope with different types of stress.^12^ Generally, this practice is adopted preventively, with the aim of strengthening the natural defenses of plants.^13^ Biostimulants are natural or synthetic compounds that contribute to growth and development processes and provide stress tolerance.^8^ Unlike conventional fertilizers, which act by directly supplying nutrients, biostimulants promote an increase in plants' ability to tolerate environmental stresses, including drought.^14^

Seaweed extracts have been extensively researched as plant biostimulants over the last few decades and are a stable source of raw material because they are renewable resources.^7^ This characteristic gives seaweed a strategic role in the development of sustainable agricultural inputs, in line with the principles of efficient use of natural resources and the promotion of sustainability in agricultural production systems.^15^

Previously reported research with different agricultural species, including tomato (*Solanum lycopersicum* L.),^16^ papaya (*Carica papaya* L.),^17^ radish (*Raphanus sativus* L.),^18^ watermelon (*Lactuca sativa* L.),^19^ and peanut (*Arachis hypogaea* L.),^11^ consistently demonstrates the effectiveness of biostimulants in mitigating abiotic stresses. These studies indicate that the application of biostimulants promotes significant improvements in physiological, biochemical, and agronomic parameters, contributing to increased plant tolerance to adverse conditions such as water deficit, salinity, and thermal stress, as well as promoting growth and productive performance in different cropping systems.

Although there have been previous studies and significant advances in recent years, the mechanisms of action of biostimulants remain partially unclear. This limitation stems largely from the high complexity of their composition, which can vary depending on the agricultural species, the origin of the biostimulant, and the soil and climate conditions of the growing region.^14^ This knowledge gap is even more evident in melon cultivation, especially when subjected to deficit irrigation in semi‐arid environments, where water and nutritional stresses often coexist.

Considering the complexity of the melon production system in semi‐arid regions, characterized by soil nutritional constraints and almost exclusive dependence on irrigation, management strategies that integrate the application of amino acid‐based biostimulants and the adoption of deficit irrigation regimes may represent a promising alternative for mitigating abiotic stresses.^20^ In this context, the study reported here aimed to evaluate the effects of the application of amino acid‐based biostimulants associated with deficit irrigation on the growth, physiological development, and productivity of yellow melons grown in semi‐arid conditions, considering three distinct climatic periods. Specifically, we sought to: (i) evaluate the effects of amino acid‐based biostimulants on stomatal conductance, leaf temperature, and chlorophyll a, chlorophyll b, and carotenoid contents; (ii) analyze melon productivity under different doses of biostimulants and levels of water availability; and (iii) evaluate the postharvest quality of melon fruits subjected to different treatments.

## MATERIALS AND METHODS

### Study area and environmental conditions

The experiment was conducted under field conditions at the Federal Rural University of Pernambuco, Serra Talhada Academic Unit (UFRPE/UAST), located in the municipality of Serra Talhada, Pernambuco, Brazil (7°57′06.0″ S and 38°17′39.2″ W, altitude of 431 m). According to the Köppen climate classification adapted for Brazil, the region presents a BSh climate, characterized as hot semi‐arid, with summer rainfall and mean annual air temperatures above 25 °C.^21^ The average annual precipitation is approximately 642.1 mm, with rainfall concentrated mainly between January and April.^22^ Meteorological data, including air temperature, relative humidity, and precipitation, were obtained from an automatic weather station operated by the National Institute of Meteorology (INMET), located approximately 100 m from the experimental area.

### Soil characterization and fertilization management

The soil of the experimental area is classified as a typical Haplic Cambisol Ta Eutrophic, presenting sandy loam texture, low acidity, and high average fertility.^23^ Prior to crop establishment, soil samples were collected from the 0–30 cm layer for fertility evaluation, following the methodology described by Chitolina *et al*.^24^ Soil correction and fertilization were performed through basal and top‐dressing applications throughout the melon cultivation cycles, according to the fertilization recommendations for the state of Pernambuco.^25^ The fertilizers used included urea, single superphosphate, potassium chloride, calcium nitrate, and magnesium sulfate.

The chemical characteristics of the soil at the beginning of each experimental period (rainy season, transition, and dry season) are presented in Table 1.

**Table 1:** Chemical attributes of the surface soil layer (0–30 cm) at the beginning of each experimental melon cultivation period in Serra Talhada, Pernambuco, UFRPE/UAST, 2023

Soil attribute	Experimental periods		
	Rainy season	Transition	Dry season
pH (H_2_O)	6.66	6.96	6.81
ECe (dS m^−1^)	0.36	4.80	3.43
MO (g kg^−1^)	15.1	7.0	23.5
P (mg dm^−3^)	79.8	108.8	55.1
S‐SO_4_^2−^ (mg dm^−3^)	2.6	25.1	24.1
Ca^2+^ (cmol_c_ dm^−3^)	4.30	4.81	8.08
Mg^2+^ (cmol_c_ dm^−3^)	1.73	1.80	4.83
K^+^ (cmol_c_ dm^−3^)	0.75	0.83	0.33
Na^+^ (cmol_c_ dm^−3^)	0.018	0.069	0.283
Al+H (cmol_c_ dm^−3^)	0.40	0.26	0.33
S (Bases) (cmol_c_ dm^−3^)	6.8	7.52	13.52
CTC (cmol_c_ dm^−3^)	7.2	7.77	13.86
Fe^2+^ (mg dm^−3^)	70.5	52.2	27.00
Mn^2+^ (mg dm^−3^)	34.8	31.3	28.6
Cu^2+^ (mg dm^−3^)	1.4	0.80	0.80
Zn^2+^ (mg dm^−3^)	1.9	3.5	0.59
B (mg dm^−3^)	0.43	1.41	0.59
V (%)	94.50	96.71	97.59

ECe: electrical conductivity of saturated paste extract; MO: organic matter; P: phosphorus content; S‐SO_4_^2−^: sulfates; Ca^2+^: calcium; Mg^2+^: magnesium; K^+^: potassium; Na^+^: sodium; Al+H: potential acidity by aluminium and hydrogen; S: sum of bases; CTC: cation exchange capacity at pH 7; Fe^2+^: iron; Mn^2+^: manganese; Cu^2+^: copper; Zn^2+^: zinc; B: boron; V: base saturation.

### Experimental design and treatments

The experiment was arranged in a randomized block design, in a 4 × 3 factorial scheme, with four replications. The treatments consisted of four application rates of an amino acid‐based biostimulant (0, 4, 6, and 8 L ha^−1^) combined with three available water (AW) levels (50%, 75%, and 100%), resulting in 12 treatments and 48 experimental units. The experiment was conducted during three distinct periods of the 2023 growing season, corresponding to the predominant climatic conditions of the region: a rainy period from 9 March to 18 May, a transition period from 9 June to 18 August, and a dry period from 1 September to 31 October. Each period corresponded to an independent crop cycle subjected to the same experimental treatments.

### Plant material and crop establishment

The plant material used was the yellow hybrid melon (*C. melo* L.) Gladial RZ F1 (Rijk Zwaan), selected due to its wide cultivation in the region, climatic adaptability, and high acceptance in the domestic market. The fruits are characterized by yellow rind, round shape, resistance to transportation, and long shelf life, with an average crop cycle of 68 to 70 days after transplanting.

**Figure 1:**
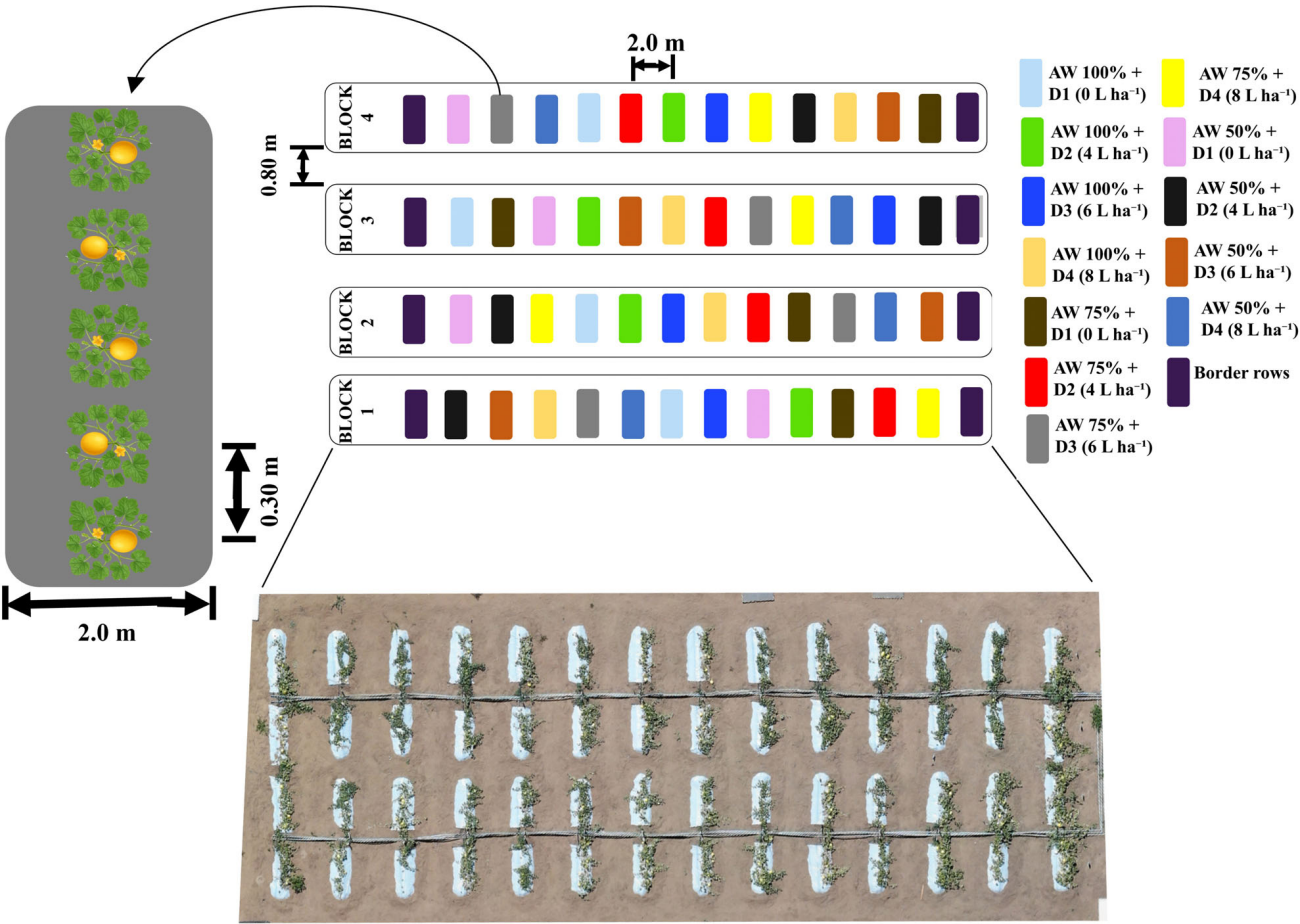
Schematic of the layout of the experimental plots and distribution of treatments for melons subjected to different amino acid application rates and irrigation depths in Serra Talhada, Pernambuco, Brazil.

Seedlings were produced in polyethylene trays with 128 cells and maintained in a greenhouse until 10 days after germination. Uniform seedlings were transplanted to raised beds covered with polyethylene mulch. Holes with a diameter of 10 cm were made in the mulch to accommodate the seedlings. The experimental area consisted of 14 planting rows, with 12 useful plots, covering a total area of 201.6 m^2^. Each plot measured 1.6 m × 2.0 m (3.2 m^2^) and contained five plants, including border plants at both ends and three central useful plants. Plant spacing was 0.3 m between plants, 2.0 m between rows, and 0.8 m between blocks, as can be seen in Fig. 1.

### Biostimulant description and application

The amino acid‐based biostimulant used in this study was Osmoplant®, a commercial formulation containing 60 g kg^−1^ free amino acids and 26.5 g kg^−1^ total nitrogen, entirely in organic form. The product is formulated with specific amino acids and osmoprotective compounds (222.1 g L^−1^), in addition to potassium and other components associated with the regulation of cellular water and osmotic balance. The application rates were defined based on the manufacturer's technical recommendation, which indicates a range of 5 to 7 L ha^−1^ for field‐grown melon crops. As no single optimal rate is specified, the selected doses (0, 4, 6, and 8 L ha^−1^) were established to include values below, within, and above the recommended range, allowing the evaluation of crop responses across different application levels. Biostimulant applications began 15 days after transplanting, with four applications performed at 10‐day intervals via fertigation, at 10, 30, 40, and 50 days after transplanting.

### Irrigation system and water management

Irrigation was performed using a drip irrigation system, with emitters spaced 0.2 m apart, a flow rate of 1.5 L h^−1^, and operating pressure of 0.9 bar. Irrigation management was based on soil moisture monitoring using CS616 sensors (Campbell Scientific®), operating on the time‐domain reflectometry principle, installed at depths of 10, 20, and 30 cm. The sensors were connected to a CR1000 datalogger powered by a 12 V, 7 Ah battery. Daily irrigation decisions were made based on sensor readings combined with continuous monitoring of meteorological conditions.

### Irrigation water quality

The irrigation water was obtained from a tubular well and classified as C_3_S_1_, indicating high salinity and low sodicity, according to Richards.^26^ The chemical characteristics of the irrigation water are presented in Table 2.

**Table 2:** Chemical analysis of irrigation water used in the melon experiment in Serra Talhada, Pernambuco, UFRPE/UAST, 2023

Property	Value
pH	6.63
EC (mS cm^−1^)	1.68
Hardness‐CaCO_3_ (mg L^−1^)	610.5
Calcium (mmol_c_ L^−1^)	5.83
Magnesium (mmol_c_ L^−1^)	6.37
Potassium (mmol_c_ L^−1^)	0.31
Sodium (mmol_c_ L^−1^)	2.04
Carbonate (mmol_c_ L^−1^)	0.00
Bicarbonate (mmol_c_ L^−1^)	3.90
Chloride (mmol_c_ L^−1^)	11.69
Sulfate (mmol_c_ L^−1^)	0.09
Boron (mg L^−1^)	0.13
Copper (mg L^−1^)	0.02
Manganese (mg L^−1^)	0.04
RAS^2/^ (mmol_c_ L^−1^)^−0,5^	0.83
RAS^3/^ (mmol_c_ L^−1^)^−0,5^	0.94

EC: electrical conductivity; RAS^2/^: sodium adsorption ratio; RAS^3/^: sodium adsorption ratio.

### Soil physical properties and irrigation scheduling

Based on soil water storage variation between field capacity (*θ*_cc_ = 0.14 cm^3^ cm^−3^) and permanent wilting point (*θ*_pmp_ = 0.01 cm^3^ cm^−3^), three irrigation depths were defined corresponding to 50% (0.07 cm^3^ cm^−3^), 75% (0.105 cm^3^ cm^−3^), and 100% (0.14 cm^3^ cm^−3^) of AW (= *θ*_cc_ − *θ*_pmp_).

The net irrigation depth (*D*_net_) was calculated using Eqn (1):

(1) $$D_{\mathrm{net}}=\mathrm{AWC}\times z$$

where *D*_net_ is the net irrigation depth (mm), AWC is the total available water capacity of the soil (mm cm^−1^), and *z* is the effective root depth (cm).

AWC was calculated according to Eqn (2):

(2) $$\mathrm{AWC}=\frac{\left(\mathrm{CC}-\mathrm{PMP}\right)}{10}\times D_{s}\times z$$

where CC is soil moisture at field capacity (% w/w), PMP is soil moisture at permanent wilting point (% w/w), and *D*_s_ is soil bulk density (g cm^−3^).

The soil physical properties are presented in Table 3.

**Table 3:** Physical characteristics of the soil in Serra Talhada, Pernambuco, UFRPE/UAST, 2023

Clean sand textural class	Clean sand
Sand (g kg^−1^)	807.6
Silt (g kg^−1^)	86.8
Clay (g kg^−1^)	105.6
*D*_p_ (g cm^−3^)	2.68
*D*_s_ (g cm^−3^)	1.41
Porosity total (cm^3^ cm^−3^)	0.48
*θ*_cc_ (cm^3^ cm^−3^)	0.14
*θ*_mpc_ (cm^3^ cm^−3^)	0.01
Hydraulic conductivity (mm h^−1^)	27.94

*D*_p_: particle density; *D*_s_: soil density.

### Soil moisture monitoring and salinity assessment

Field capacity was determined *in situ* following EMBRAPA,^27^ while the permanent wilting point was determined using the physiological humid chamber method proposed by Briggs and Shantz,^28^ adapted by EMBRAPA.^27^ Sensor calibration was performed in the laboratory to adjust dielectric constants, following the manufacturer's guidelines.

After each harvest, soil samples from the 0–30 cm layer of each plot were collected to evaluate soil salinity through electrical conductivity of the saturation extract (ECe), following Teixeira *et al*.^29^ Approximately 200 g of soil was sieved, saturated with distilled water, and stored for 24 h. The soil solution was extracted using vacuum suction and analyzed with a calibrated bench conductivity meter, with results expressed in mS cm^−1^.

### Physiological measurements

Physiological evaluations were performed every 10 days prior to harvest. Stomatal conductance (gs) was measured using a portable porometer (SC‐1 Leaf Porometer), and leaf temperature (LT) was recorded using an infrared thermometer directed at the adaxial surface from a minimum distance of 10 cm. Measurements were taken between 10:00 and 11:00 and 14:00 and 15:00 on the same leaf of the central plant in each plot.

For chlorophyll analysis, one leaf per plant was collected during the week preceding harvest. Samples were wrapped in aluminium foil, stored in a thermal container with ice, frozen in liquid nitrogen, and kept at −80 °C. Leaf tissue (0.2 g) was macerated and extracted with 10 mL of 80% acetone under dark conditions. Absorbance readings were obtained using a UV–visible spectrophotometer, and chlorophyll a, chlorophyll b, and carotenoids were calculated following Lichtenthaler and Buschmann,^30^ using Eqns ((3), (4), (5))–((3), (4), (5)):

(3) $$C_{a}\;\left(\mathrm{\mu g}\;{\mathrm{mL}}^{-1}\right)=12.25A_{663.2}-2.79A_{646.8}$$

(4) $$C_{b}\;\left(\mathrm{\mu g}\;{\mathrm{mL}}^{-1}\right)=12.50A_{646.8}-5.10A_{663.2}$$

(5) $$C_{\left(x+c\right)}\;\left(\mathrm{\mu g}\;{\mathrm{mL}}^{-1}\right)=\frac{\left(1000A_{470}-1.82C_{a}-85.02C_{b}\right)}{198}$$

### Fruit yield and quality attributes

Fruit harvest was carried out when the fruits reached commercial maturity, defined by external coloration and physiological development characteristic of the cultivar. During the rainy and transition seasons, harvest occurred at 70 days after transplanting (DAT), while in the dry season it was performed at 67 DAT. All fruits from the useful area of each plot were harvested manually.

After harvest, fruits were individually counted and weighed using a precision digital scale to determine fruit yield per plot, which was later converted to yield on an area basis (Mg ha^−1^). Fruit biometric characteristics were assessed by measuring equatorial and longitudinal diameters using a graduated measuring tape, ensuring consistent positioning for all samples.

Fruit firmness was evaluated in the laboratory using a computerized universal testing machine with a maximum load capacity of 100 kg (model IP‐90). For rind firmness, an 8 mm diameter cylindrical probe was used to apply force perpendicularly to the fruit surface at three distinct regions: equatorial region, near the blossom end, and near the stem end. Each measurement was performed in triplicate, and the average value was recorded. Pulp firmness was determined using the same equipment and probe, applying force until tissue rupture occurred after rind penetration.

Total soluble solids content was determined using juice extracted from the fruit pulp. Approximately 1 mL of juice was obtained from each fruit and analyzed using a digital bench refractometer (Instrutherm®, model RTD‐95, São Paulo, Brazil), previously calibrated with distilled water. Results were expressed in degrees Brix (°Brix), following previously described analytical procedures.^31^

Titratable acidity (TA) was determined by acid–base titration. A 10 mL aliquot of juice extracted from the fruit pulp was titrated with standardized 0.1 N sodium hydroxide (NaOH) solution, using phenolphthalein as an indicator to identify the titration endpoint. The results were calculated according to Eqn (6) and expressed as a percentage of citric acid, which is the predominant organic acid in melon fruits:

(6) $$\mathrm{AT}=\frac{N_{\text{NaOH}}\cdot V_{\text{NaOH}}\cdot {\mathrm{Eq}}_{\mathrm{ac}}}{V_{\mathrm{suc}}}$$

In addition, relative productivity (Pr) was calculated to evaluate the impact of soil salinity on crop performance. Relative productivity values were obtained by relating the actual productivity of each treatment to the maximum productivity observed under optimal water availability conditions (100% of AW). The ECe measured at the end of each cultivation cycle was used as the reference indicator of soil salinity. Relative productivity was calculated according to Eqn (7) and expressed as a percentage:

(7) $$\mathrm{Pr}\;\left(\%\right)=\frac{\mathrm{PRe}}{\mathrm{PM}}\times 100$$

### Statistical analysis

Data were tested for normality and homogeneity of variances using the Shapiro–Wilk and Levene tests, respectively. The experiment was conducted using a two‐factor factorial design, in which the factors and their respective levels were evaluated simultaneously. Data were subjected to analysis of variance considering the main effects of the factors and their interaction. When significant effects were detected, means of qualitative factors were compared using Tukey's test at the 5% probability level. Quantitative factors were analyzed by regression analysis, selecting the model that best fitted the data based on the significance of regression coefficients and the coefficient of determination (*R* ^2^). All statistical analyses were performed using R software (version 4.2.0), and graphs were generated using SigmaPlot (version 14.0).

## RESULTS

### Climatic conditions and soil moisture

**Figure 2:**
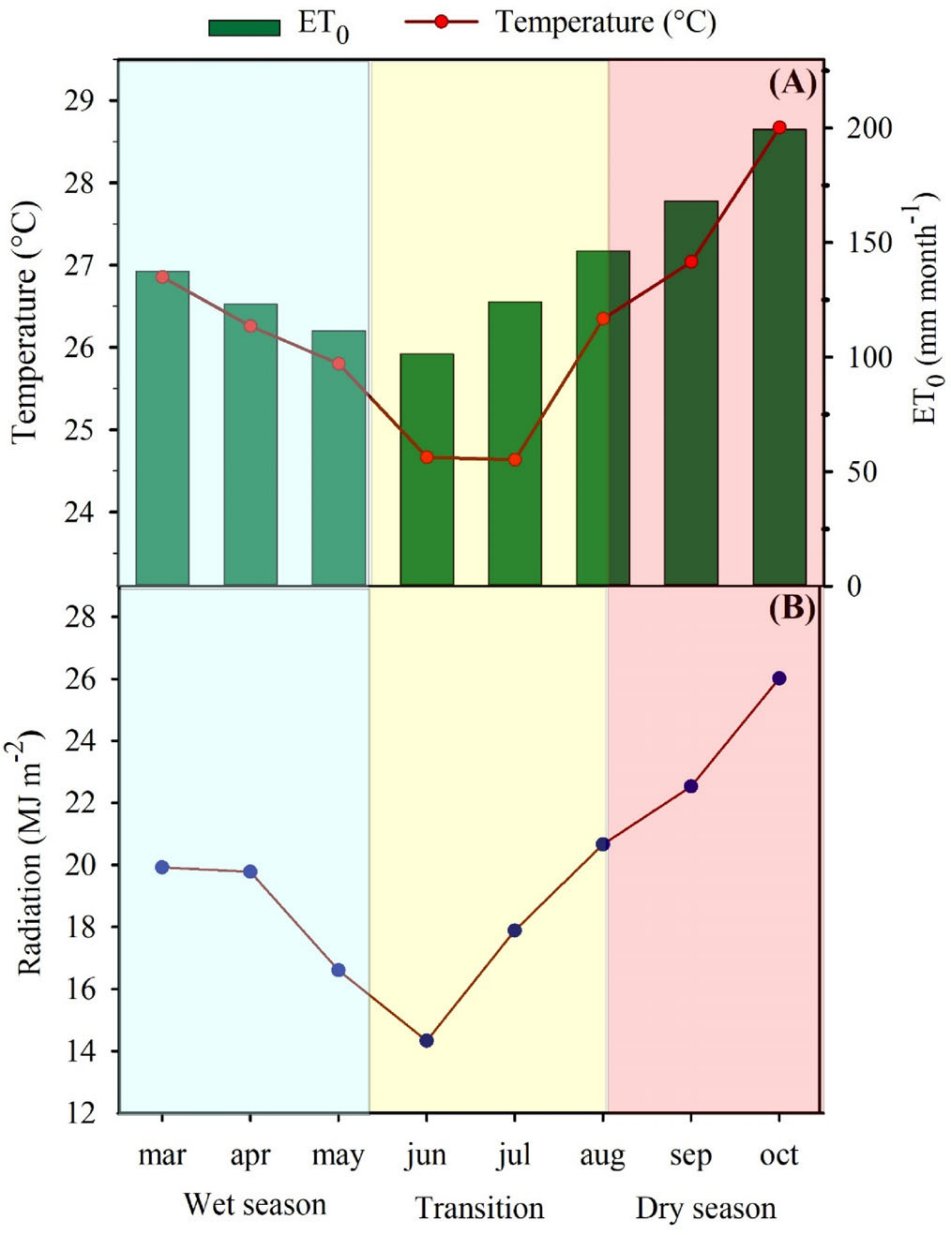
Average climatic condition in Serra Talhada, Pernambuco area (wet season, transition period and dry season). (A) Temperature shown by lines and reference evapotranspiration ET_0_. (B) Solar radiation.

Climatic conditions varied substantially among the cultivation periods, influencing atmospheric demand and soil water dynamics throughout the experiment. During the rainy season (March–May), mean air temperature ranged from approximately 26.8 to 25.8 °C, accompanied by a progressive reduction in reference evapotranspiration (ET_0_), which declined from about 137 to 111 mm month^−1^, along with decreasing solar radiation. These conditions resulted in moderate evaporative demand and relatively high air humidity (Fig. 2).

The transition period (June to mid‐August) was marked by the lowest climatic demand of the study. Mean air temperature remained close to 24.6 °C, while ET₀ varied approximately between 101 and 146 mm month^−1^ and solar radiation ranged from about 14.3 to 20.7 MJ m^−2^. In contrast, during the dry season (late August–October), air temperature increased from roughly 26.3 to 28.7 °C, accompanied by higher ET₀ (about 146 to 199 mm month^−1^) and increased solar radiation, reaching values close to 26 MJ m^−2^, together with a marked reduction in relative humidity.

**Figure 3:**
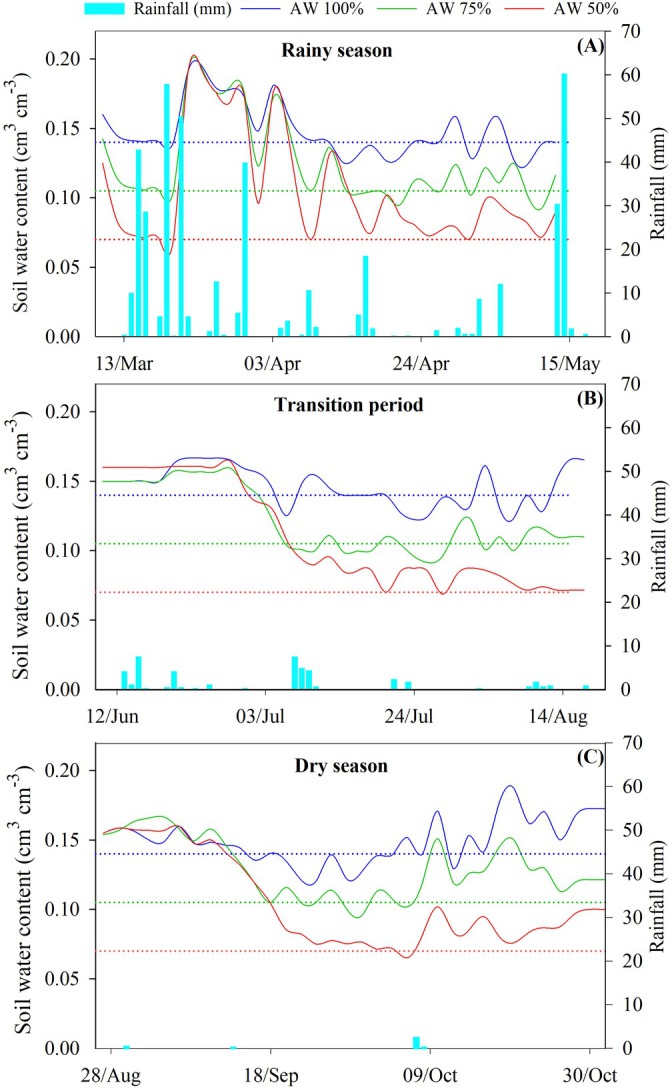
Monitoring of soil water content and rainfall during the rainy season (A), the transition period (B), and the dry season (C) for the experimental melon crop in 2023 in Serra Talhada, Pernambuco. Dotted horizontal lines indicate the soil water content limits corresponding to each level of availability (50%, 75%, and 100% AW).

Rainfall distribution played a decisive role in soil moisture behavior. During the rainy season, precipitation was concentrated in the first 2 weeks after transplanting, totaling approximately 199 mm, followed by lower but regular rainfall throughout the cycle, with an additional accumulation of about 222 mm and a final precipitation event of 93 mm, resulting in a total of approximately 422 mm during the cultivation period. This rainfall pattern maintained soil water content above field capacity across all irrigation treatments for about 4 weeks, delaying the differentiation among water regimes until the fifth week of cultivation. During the transition period, rainfall declined sharply to approximately 44 mm, representing a reduction of nearly 90% compared with the rainy season, which led to an earlier separation of soil moisture levels among treatments from the third week after transplanting. In the dry season, precipitation was minimal (about 3 mm), corresponding to a reduction of more than 99% relative to the rainy period, resulting in rapid soil moisture depletion and a clear differentiation among irrigation treatments throughout most of the cultivation cycle (Fig. 3).

Irrigation management based on depletion of AW generated distinct water supply patterns among treatments and cultivation periods. During the transplant establishment phase, irrigation was applied uniformly across all treatments for approximately 10 days to ensure plant adaptation. Thereafter, irrigation depths were adjusted according to soil moisture thresholds corresponding to 50%, 75%, and 100% AW. Total accumulated water input declined sharply from the rainy season to the transition and dry seasons for all treatments. Under the 50% AW regime, accumulated water supply decreased from approximately 430 mm in the rainy season to about 55 and 17 mm in the transition and dry seasons, respectively, reflecting reductions of nearly 87% and 96%. Similar declining trends were observed for the 75% and 100% AW treatments across cultivation periods (Table 4).

**Table 4:** Parameters of irrigation controlled by depletion of AW content at 50%, 75%, and 100%

Period	ET_0_ (mm)	Rainfall (mm)	Irrigation depth (mm)^a^		
			50% AW	75% AW	100% AW
	*Rainy season*				
9–15 March	34.59	53.20	7.62	7.62	7.62
16–22 March	24.91	146.20	0.00	0.00	0.00
23–29 March	30.82	19.60	0.00	2.54	2.54
30 March–5 April	27.12	45.40	0.00	0.00	0.00
6–12 April	28.26	13.20	0.00	0.00	0.00
13–19 April	30.34	25.40	0.00	2.11	16.26
20–26 April	27.69	1.80	0.33	12.70	24.60
27 April–3 May	27.27	11.80	0.00	2.41	10.23
4–10 May	27.39	12.00	0.00	0.83	14.28
11–18 May	29.20	93.00	0.00	3.82	7.54
**Total**	**287.60**	**421.60**	**7.95**	**32.01**	**83.09**
	*Transition period*				
9–15 June	24.95	12.40	7.62	7.62	7.62
16–22 June	22.78	5.00	2.54	2.54	2.54
23–29 June	23.64	1.20	0.00	0.00	0.00
30 June–6 July	25.67	0.20	0.00	0.44	4.46
7–13 July	22.10	17.00	0.00	3.40	14.07
14–20 July	30.32	0.00	0.00	1.49	7.76
21–27 July	30.14	3.80	0.00	3.68	10.56
28 July–3 August	31.27	0.20	0.40	2.72	2.61
4–10 August	34.00	2.20	0.00	1.26	7.63
11–18 August	35.74	2.20	0.00	0.00	8.44
**Total**	**280.60**	**44.20**	**10.56**	**23.14**	**65.69**
	*Dry season*				
26 August–1 September	35.49	0.40	10.15	10.15	10.15
2–8 September	37.72	0.00	2.53	2.53	2.53
9–15 September	38.14	0.20	0.00	1.26	6.59
16–22 September	37.83	0.00	0.00	2.38	9.37
23–29 September	42.39	0.00	0.00	1.41	11.54
30 September–6 October	45.37	0.00	1.58	9.29	18.23
7–13 October	41.60	2.60	0.00	1.26	9.53
14–20 October	44.93	0.00	0.00	4.31	11.42
21–31 October	80.13	0.00	0.00	0.00	0.00
**Total**	**403.6**	**3.2**	**14.26**	**32.59**	**79.36**

^a^ Replacement of irrigation depths from critical levels of soil water content equal to 0.07, 0.105, and 0.14 cm^3^ cm^−3^ for 50%, 75%, and 100% AW, respectively.

For the 75% AW treatment, irrigation was withheld during the rainy season in the second, fourth, and fifth weeks of cultivation, as soil moisture remained above the critical threshold (*θ* = 0.105 cm^3^ cm^−3^). When soil water content dropped below this limit, irrigation was applied only to restore moisture to the critical level, resulting in a total irrigation depth of 32.01 mm. During the transition period, irrigation was omitted in the third and tenth weeks, leading to an accumulated irrigation of 23.14 mm. In the dry season, irrigation was suspended in the final 2 weeks of cultivation, totaling 32.59 mm. Considering rainfall contributions, the cumulative water input reached 453.61, 63.34, and 35.79 mm for the rainy, transition, and dry seasons, respectively, representing reductions of 86.03% and 92.10% from the rainy season to the transition and dry periods.

For the 100% AW depth, during the rainy season, irrigation was omitted in the second, fourth, and fifth weeks because soil moisture remained above the critical point (*θ* = 0.14 cm^3^ cm^−3^), equivalent to field capacity. When soil moisture dropped below this threshold, irrigation was applied, resulting in a total of 83.09 mm of irrigation water. In the transition period, irrigation was withheld in the third week due to adequate soil moisture, leading to an accumulated irrigation depth of 65.69 mm. In the dry season, irrigation was not performed in the final 2 weeks, totaling 79.36 mm. Considering rainfall, the total water input for the 100% AW treatment reached 504.69, 109.89, and 82.56 mm in the rainy, transition, and dry seasons, respectively, corresponding to reductions of 78.22% and 83.64% relative to the rainy season.

### Soil salinity

Soil salinity exhibited distinct temporal patterns throughout the cultivation periods, reflecting the contrasting rainfall distribution and irrigation regimes described in the previous section, as well as differences in irrigation depths imposed by the AW levels (Table 5). Variations in ECe were associated with both seasonal water availability and irrigation management, with clear differences among AW treatments that became more pronounced as climatic conditions shifted across cultivation periods.

**Table 5:** Measurements of ECe in dS m^−1^ in the experiment using the soil saturation paste extraction method in Serra Talhada, Pernambuco, UFRPE/UAST, 2023

Factor	Treatment	Rainy season	Transition	Dry season
Irrigation (% AW)	50	1.26ab	4.88	4.63
	75	1.04b	5.64	5.00
	100	1.42a	5.89	4.54
Doses (L ha^−1^)	0	1.35	4.67	4.91
	4	1.29	6.03	4.80
	6	1.03	5.81	4.42
	8	1.29	5.37	4.75
Interaction (I × D)		ns	ns	ns
CV (%)		27.57	34.31	35.03

Within each column, values followed by the same letter are not significantly different at *P* < 0.05; ns, non‐significant interaction.

During the rainy season, significant differences in soil salinity were observed between the different irrigation depths (Table 5). However, no effect of the biostimulant doses was observed, nor was there any interactive effect between the two factors studied (Table 5). Despite the high and well‐distributed rainfall described in Climatic conditions and soil moisture section, soils receiving 100% AW exhibited higher ECe compared with the 50% and 75% AW treatments (Table 5). This pattern indicates that, under rainy season conditions, irrigation depth influenced the distribution of salts in the soil profile, even in the presence of substantial natural leaching promoted by rainfall.

As rainfall decreased sharply after the wet season, soil salinity rose during the transition period and remained elevated throughout the cultivation cycle. ECe reached values between 5.37 and 6.03 dS m^−1^ (Table 5), regardless of irrigation depth or fertigation rate. The absence of statistical differences among treatments suggests that, under transitional climatic conditions, seasonal constraints dominated salinity dynamics, reducing the relative influence of irrigation management.

During the dry season, soil salinity remained high across all treatments, with ECe values ranging from 4.42 to 4.91 dS m^−1^ (Table 5), comparable to those observed during the transition period. Despite the pronounced reduction in rainfall and increased evaporative demand described in Climatic conditions and soil moisture section, no significant differences were detected among irrigation depths or fertigation rates. These results indicate that, under dry‐season conditions, the overall water limitation outweighed the effects of the irrigation and fertigation treatments on soil salinity.

Under dry‐season conditions, soil salinity remained high, although with a slight reduction compared to the transition period. ECe values ranged from 4.42 to 4.91 dS m^−1^ (Table 5), indicating that even with lower water inputs, salt accumulation persisted in the root zone. The lack of significant differences among irrigation depths suggests that limited rainfall and high evaporative demand constrained salt leaching, making seasonal climatic conditions the primary driver of salinity behavior during this period.

However, fertigation rates did not significantly affect soil salinity in any of the cultivation periods (Table 5). Electrical conductivity values remained statistically similar across application rates ranging from 0 to 8 L ha^−1^, indicating that the contribution of fertigation to salt buildup was secondary compared to climatic seasonality and irrigation regime. The absence of interaction between irrigation depth and fertigation rate further reinforces that soil salinity dynamics were predominantly governed by environmental factors rather than management‐induced salt inputs.

### Stomatal conductance, leaf temperature, and chlorophyll a, chlorophyll b, and carotenoid contents

The patterns of soil salinity observed across the cultivation periods (Soil salinity section) provide an essential context for understanding the physiological responses of melon plants. Variations in ECe associated with irrigation depth, seasonal water availability, and fertigation regimes created distinct environmental conditions that likely influenced stomatal behavior, LT, and pigment contents. Elevated soil salinity during the transition and dry seasons may have imposed osmotic stress, affecting water uptake and gas exchange, whereas more moderate salinity levels in the rainy season allowed near‐optimal physiological activity. Within this environmental framework, gs, LT, and chlorophyll and carotenoid contents were evaluated to determine how irrigation and biostimulant treatments modulated plant performance under differing soil salinity conditions.

The gs and LT showed distinct responses depending on the cultivation period, irrigation depth, and biostimulant rate (Table 6). During the rainy season, significant differences in gs were observed only in the afternoon (14:00–15:00). Plants irrigated at 100% AW exhibited higher gs (288.31 mmol m^−2^ s^−1^) than those under 50% AW (206.68 mmol m^−2^ s^−1^) and 75% AW (226.31 mmol m^−2^ s^−1^), while LT remained statistically similar across treatments (Table 6). These results indicate that sufficient water availability promotes maximal stomatal opening, facilitating gas exchange, whereas LT is less sensitive to moderate differences in water supply during wet conditions.

**Table 6:** Effect of amino acid‐based biostimulant rate and irrigation depths on gs (mmol m^−2^ s^−1^) and LT (°C) of melon plants

Factor	Treatment	Rainy season			
		gs (10:00–11:00)	LT (10:00–11:00)	gs (14:00–15:00)	LT (14:00–15:00)
Irrigation (% AW)	50	259.01	35.40	206.68b	35.50
	75	236.43	36.43	226.31b	34.55
	100	279.68	36.78	288.31a	35.02
Doses (L ha^−1^)	0	267.40	35.73	244.13	35.40
	4	241.09	36.45	236.62	35.12
	6	256.77	36.00	246.52	35.06
	8	268.23	36.62	234.46	34.50
Interaction (I × D)		ns	ns	ns	ns
CV (%)		23.78	6.07	17.58	5.26

Factor	Treatment	Transition			
		gs (10:00–11:00)	LT (10:00–11:00)	gs (14:00–15:00)	LT (14:00–15:00)
Irrigation (% AW)	50	196.43	26.15	235.79	33.51
	75	227.25	26.15	229.04	32.71
	100	206.45	27.11	221.13	33.69
Doses (L ha^−1^)	0	215.24	27.35	210.00	32.08
	4	226.86	26.27	236.26	32.95
	6	208.18	27.71	249.39	33.87
	8	189.90	26.43	218.96	34.32
Interaction (I × D)		ns	ns	ns	ns
CV (%)		29.76	15.92	26.51	8.39

Factor	Treatment	Dry season			
		gs (10:00–11:00)	LT (10:00–11:00)	gs (14:00–15:00)	LT (14:00–15:00)
Irrigation (% AW)	50	144.62 a	34.93	126.91a	39.78ab
	75	112.59 b	35.99	131.65a	38.02b
	100	86.11b	37.32	91.38b	40.62a
Doses (L ha^−1^)	0	125.95	36.50	139.6	39.09
	4	125.80	34.50	119.15	39.18
	6	113.05	37.53	107.9	40.05
	8	92.95	35.80	99.95	39.58
Interaction (I × D)		ns	ns	ns	ns
CV (%)		30.93	8.83	28.23	6.68

Within each column, values followed by the same letter are not significantly different at *P* < 0.05; ns, non‐significant interaction.

In the transition period, no significant differences in gs or LT were detected among irrigation levels or biostimulant rates (Table 6). This uniformity likely reflects moderate environmental conditions, where neither water deficit nor high evaporative demand strongly affected plant physiological behavior. The absence of significant variation underlines the resilience of melon plants to intermediate seasonal conditions and highlights that stomatal and thermal responses are more pronounced under extreme environmental stress.

LT largely reflected environmental conditions rather than treatment effects. No significant differences were observed in the rainy and transition periods for either irrigation or biostimulant rates (Table 6). However, in the dry season, afternoon LT was higher in plants receiving 75% AW compared to 50% and 100% AW, which were statistically similar. Interestingly, the minimum LT across cultivation periods was generally observed in plants without biostimulant (0 L ha^−1^), suggesting that biostimulant application may subtly influence leaf thermal regulation under water‐limited conditions (Table 6).

The dry season presented a contrasting picture. In the morning (10:00–11:00), gs was higher for 50% AW (144.62 mmol m^−2^ s^−1^), exceeding the values observed for 75% (112.59 mmol m^−2^ s^−1^) and 100% AW (86.11 mmol m^−2^ s^−1^). However, there was no significant effect of the applied biostimulant doses (*P* > 0.05) (Table 6).

**Figure 4:**
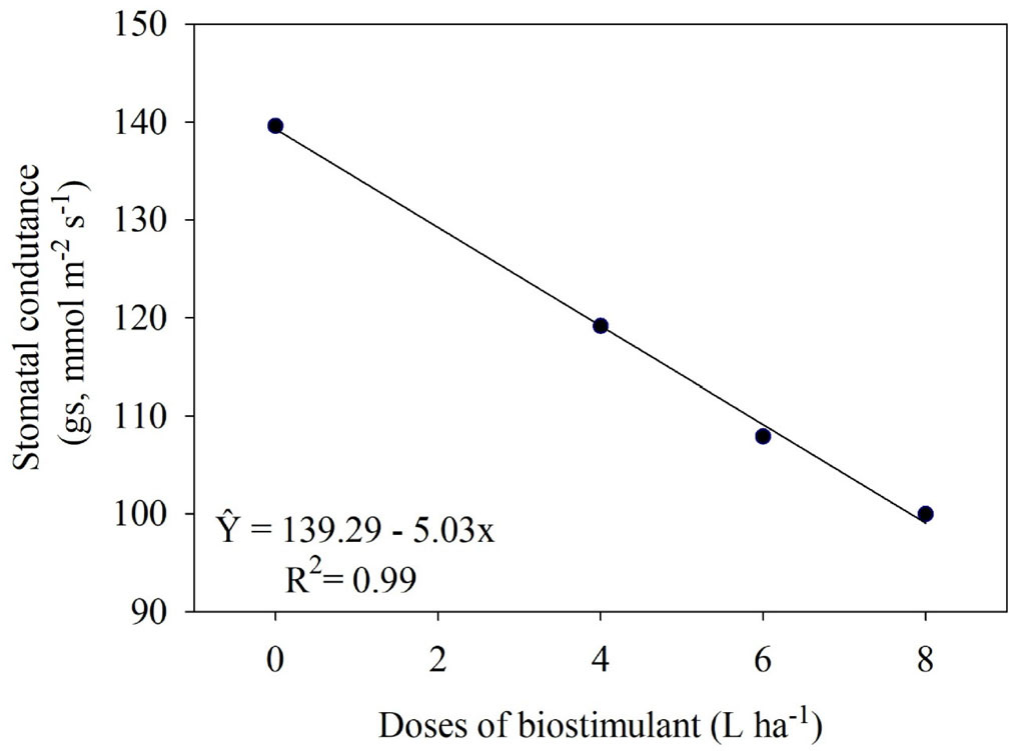
Values of gs measured at 14:00–15:00 during the dry season as a function of biostimulant doses.

As for the afternoon measurements (14:00–15:00), there was a significant effect of the doses applied (*P* < 0.05) (Fig. 4). In this case, a linear relationship was observed, whereby gs decreased as the doses of biostimulants increased (Fig. 4).

Plants without biostimulant application (0 L ha^−1^) exhibited the highest gs (139.6 mmol m^−2^ s^−1^), while the highest application rate (8 L ha^−1^) produced the lowest conductance (99.95 mmol m^−2^ s^−1^) (Fig. 4). The intermediate doses (4 and 6 L ha^−1^) resulted in gs values of 119.15 and 107.9 mmol m^−2^ s^−1^, respectively (Fig. 4). This trend indicates that increasing doses of biostimulants tend to reduce gs under dry season stress, with the most pronounced effect observed at the highest dose.

Chlorophyll a, chlorophyll b, and carotenoid contents were not significantly affected by irrigation depth or biostimulant rate within each cultivation period (Table 7). Nonetheless, seasonal trends were evident: chlorophyll concentrations were higher in the rainy and transition periods than in the dry season, reflecting the negative impact of water and salinity stress on pigment synthesis and stability. Carotenoid contents also varied seasonally, generally decreasing under dry conditions, which may indicate stress‐induced limitations in pigment accumulation. Despite the lack of statistically significant treatment effects, these seasonal patterns highlight the influence of environmental conditions on plant physiological status. Furthermore, no interactive effect was observed between the doses of biostimulants applied and the irrigation depths.

**Table 7:** Chlorophyll a, chlorophyll b, and carotenoid contents (μg ml^−1^) in melon crops in Serra Talhada, Pernambuco, UFRPE/UAST, 2023

Factor	Treatment	Chlorophyll a	Chlorophyll b	Carotenoids
		*Rainy season*		
Irrigation (% AW)	50	17.54	8.66	4.26
	75	17.18	8.70	4.47
	100	12.32	6.41	3.63
Doses (L ha^−1^)	0	14.86	7.29	4.02
	4	14.12	7.30	4.02
	6	15.15	7.77	3.91
	8	18.61	9.32	4.52
Interaction (I × D)		ns	ns	ns
CV (%)		43.06	39.92	26.13
		*Transition period*		
Irrigation (% AW)	50	16.01	7.67	4.64
	75	13.51	6.41	5.64
	100	12.44	6.06	4.51
Doses (L ha^−1^)	0	14.76	6.73	4.70
	4	13.02	6.38	4.35
	6	13.31	6.29	4.18
	8	14.86	7.46	4.62
Interaction (I × D)		ns	ns	ns
CV (%)		33.21	30.27	23.84
		*Dry season*		
Irrigation (% AW)	50	11.53	5.87	4.21
	75	14.68	7.05	4.47
	100	11.53	5.50	4.33
Doses (L ha^−1^)	0	13.51	6.45	4.15
	4	11.96	5.63	4.42
	6	11.91	6.05	4.54
	8	13.14	6.43	4.24
Interaction (I × D)		ns	ns	ns
CV (%)		35.24	34.51	35.03

ns, non‐significant interaction.

Overall, gs and LT were influenced primarily by irrigation level and environmental conditions, with biostimulant application showing effects mainly under dry‐season stress. Chlorophyll and carotenoid contents varied more between cultivation periods than among treatments, suggesting that seasonal water availability and soil salinity were stronger drivers of pigment dynamics than irrigation or biostimulant application. These findings provide insight into the physiological responses of melon plants under varying water and salinity conditions, which may inform irrigation and biostimulant management strategies.

### Postharvest

The postharvest characteristics of melon fruits were influenced by irrigation level, biostimulant rate, and seasonal variation, with distinct patterns observed across the three cultivation periods (Table 8). These traits, including the number of fruits per plant, fruit weight, dimensions, pulp and skin firmness, total soluble solids, and TA, provide insight into the effects of water management and biostimulant application on fruit quality.

**Table 8:** Average values of number of fruits per plant (NF), fruit weight (FW, kg), equatorial diameter (DE, mm), longitudinal diameter (DL, mm), skin firmness (FC, N), pulp firmness (PF, N), total soluble solids (TSS, °Brix), and titratable acidity (TA, %) in melon fruits across three cultivation periods

Fator	Trat.	NF	FW	DE	DL	FC	PF	TSS	TA
		*Rainy season*							
Irrigation (% AW)	50	3.25b	1.26b	14.33b	16.29b	64.05	16.54	12.23	0.63
	75	4.12ab	1.61a	15.34a	17.62a	61.03	15.11	12.98	0.68
	100	4.75a	1.37ab	14.69ab	16.62b	63.50	17.31	12.65	0.66
Doses (L ha^−1^)	0	3.41	1.47	14.71	16.79	63.33	17.64	12.58	0.63
	4	4.58	1.31	14.49	16.60	64.12	18.41	12.37	0.64
	6	4.58	1.33	14.85	17.00	61.88	14.57	12.62	0.66
	8	3.58	1.55	15.09	16.95	62.11	14.64	12.90	0.69
Interaction (I × D)		ns	ns	ns	ns	ns	ns	ns	ns
CV (%)		26.05	22.57	6.09	5.95	7.58	19.67	7.82	12.5
		*Transition period*							
Irrigation (% AW)	50	2.18	0.55	9.90	10.61	59.27	10.47	13.01	0.58
	75	2.43	0.64	10.38	11.18	61.68	10.51	11.35	0.56
	100	2.37	0.70	10.84	11.76	64.24	10.34	11.60	0.50
Rate (L ha^−1^)	0	2.33	0.62	10.31	11.18	61.93	10.25	13.55	0.51
	4	2.50	0.64	10.34	11.14	61.95	9.81	12.10	0.48
	6	2.16	0.68	10.38	11.34	61.79	10.75	11.29	0.64
	8	2.33	0.57	10.42	1109	62.55	10.87	11.01	0.56
Interaction (I × D)		ns	ns	ns	ns	ns	ns	ns	ns
CV (%)		28.08	29.85	9.24	9.46	16.07	18.06	18.00	35.66
		*Dry season*							
Irrigation (% AW)	50	0.93	0.35	10.00	10.25b	60.92	18.74a	10.13	1.37
	75	0.97	0.45	10.13	10.78ab	60.20	12.19b	11.25	1.47
	100	1.02	0.56	10.66	11.59a	59.20	20.06a	10.55	1.38
Rate (L ha^−1^)	0	0.77	0.45	10.38	10.96	60.13	16.73	10.79	1.50
	4	0.83	0.50	10.64	11.34	61.15	17.24	10.94	1.40
	6	0.72	0.41	9.93	10.52	58.98	20.06	10.36	1.35
	8	0.77	0.47	10.08	10.67	60.16	19.55	10.49	1.37
Interaction (I × D)		ns	ns	ns	ns	ns	ns	ns	ns
CV (%)		30.71	31.1	9.48	9.68	8.31	16.71	19.37	15.43

Means followed by the same letters in the columns do not differ from each other by Tukey's test at 5% probability; ns: non‐significant interaction; Trat.: treatments.

During the rainy season, irrigation at 100% AW resulted in the highest number of fruits per plant (4.75) and average fruit weight (1.61 kg), whereas 50% AW produced the lowest values (3.25 fruits per plant and 1.26 kg). Among biostimulant treatments, the 4 L ha^−1^ rate was notable for promoting higher pulp firmness (18.41 N) and elevated total soluble solids (12.90 °Brix). No significant interactions between irrigation and biostimulant rate were observed for any trait, indicating that each factor independently influenced fruit characteristics during this period.

In the transition period, the overall fruit production and weight decreased compared to the rainy season, reflecting the influence of reduced water availability and environmental stress. Although irrigation at 100% AW generally produced slightly higher fruit quality parameters, differences among irrigation levels were not statistically significant. Total soluble solids were highest in the untreated control (0 L ha^−1^, 13.55 °Brix), but no significant differences were detected between biostimulant rates or in combination with irrigation treatments. This pattern suggests that fruit quality during the transition period is more strongly constrained by seasonal conditions than by management factors.

During the dry season, the reduction in fruit number and weight was again evident across treatments. Fruits irrigated at 100% AW maintained slightly better pulp firmness and total soluble solids, although differences among irrigation levels were not significant. Among biostimulant rates, the 6 L ha^−1^ treatment produced the highest pulp firmness, indicating a potential positive effect of moderate biostimulant application under stress conditions. Across both the transition and dry periods, no significant differences in pulp firmness were observed with irrigation level, while reductions in firmness compared to the rainy season were noted. Specifically, average pulp firmness decreased by 5.88 N in the transition period compared to the rainy season and by 6.57 N when considering biostimulant treatments, highlighting the impact of seasonal environmental stress on fruit texture.

Total soluble solids andTA were relatively stable across treatments within each period. No significant effects of irrigation or biostimulant rate were observed, although slight variations occurred between periods. TA tended to be higher in the rainy season, decreasing slightly in the dry season, suggesting that environmental conditions, rather than management practices, primarily influenced these postharvest quality parameters.

Overall, melon fruit characteristics were influenced primarily by seasonal environmental conditions rather than by irrigation level or biostimulant rate. While irrigation at 100% AW and moderate biostimulant rates (4–6 L ha^−1^) showed trends toward higher fruit number, weight, and pulp firmness, most differences were not statistically significant. These findings are consistent with the physiological responses observed in Stomatal conductance, leaf temperature, and chlorophyll a, chlorophyll b, and carotenoid contents section, illustrating how environmental factors such as water availability and soil salinity translate into fruit‐level outcomes.

**Figure 5:**
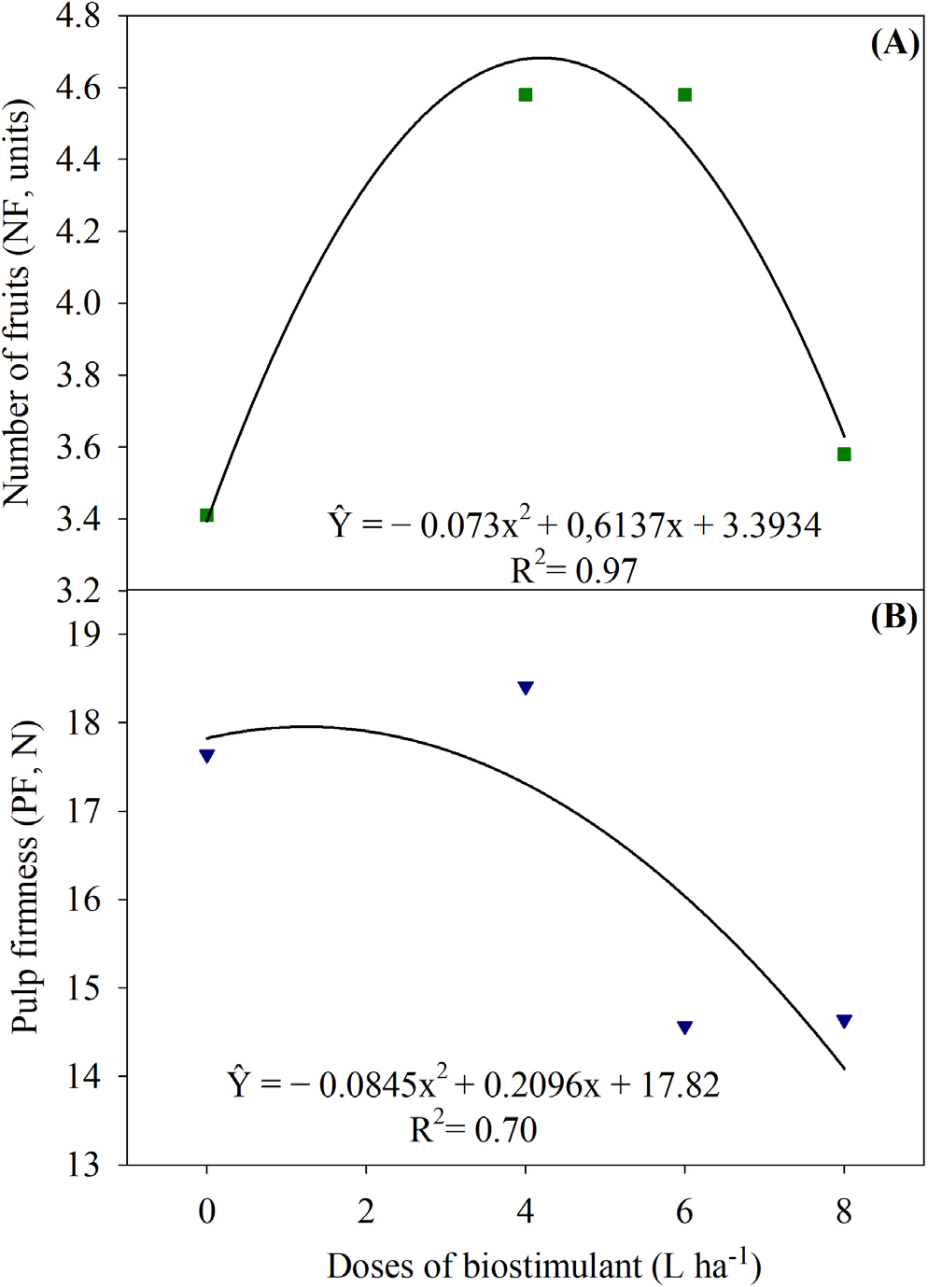
Effect of biostimulant doses on melon fruit characteristics. (A) Number of fruits per plant and (B) pulp firmness. Points represent treatment means and lines correspond to fitted regression models.

Biostimulant doses significantly affected the number of fruits per plant and pulp firmness of melon fruits (Fig. 5). The number of fruits per plant showed a quadratic response to increasing biostimulant doses (Fig. 5). Fruit production increased with the application of the product up to intermediate doses, reaching maximum values near the 4 L ha^−1^ treatment. At higher doses, a reduction in fruit number was observed, indicating a decline in plant productivity at elevated application rates.

Pulp firmness was also significantly influenced by biostimulant doses, presenting a quadratic response (Fig. 5). Higher firmness values were observed at lower doses, while a progressive reduction occurred with increasing application rates. The lowest firmness values were recorded at the highest biostimulant doses. Overall, the results indicate that intermediate doses of the biostimulant promoted higher fruit production, whereas excessive doses negatively affected fruit quality attributes such as pulp firmness.

### Productivity and water use efficiency

**Figure 6:**
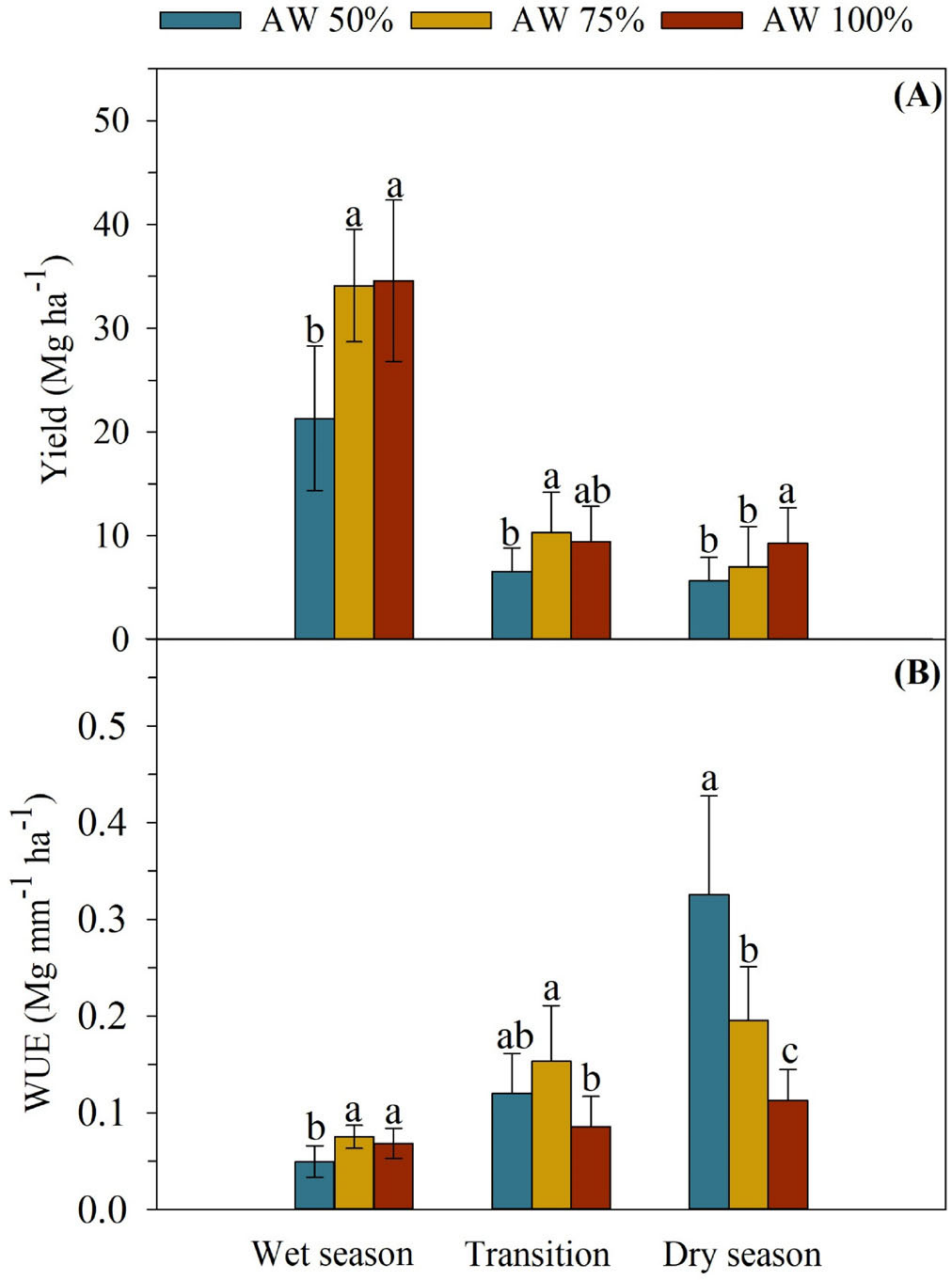
Yield (A) and WUE (B) of melon plants grown under irrigation levels corresponding to 50%, 75%, and 100% AW during three cultivation periods in Serra Talhada, Pernambuco, Brazil, in 2023.

**Figure 7:**
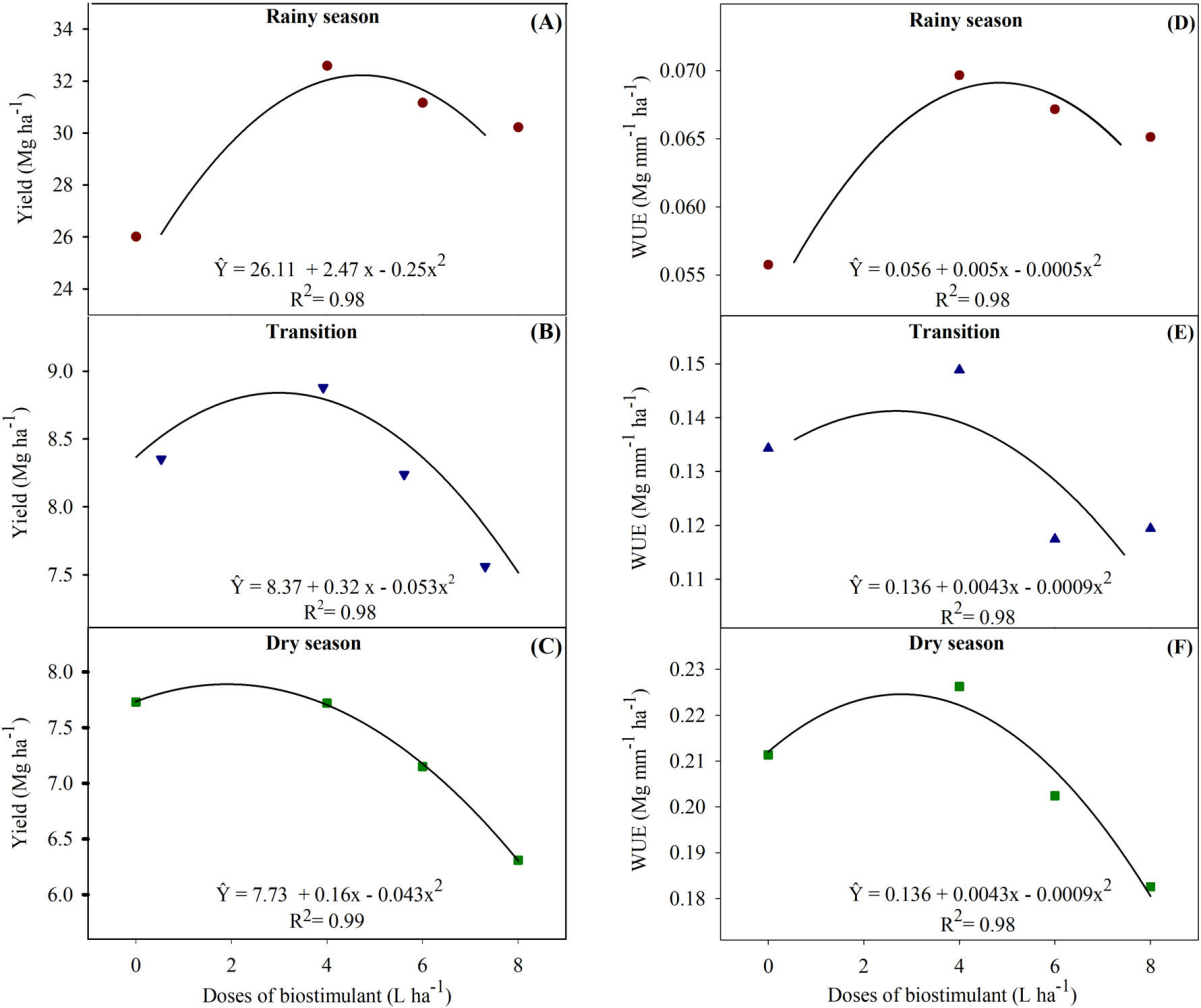
(A–C) Yield and (D–F) WUE of melon as affected by biostimulant application rates (0, 4, 6, and 8 L ha^−1^) across three cultivation cycles conducted in Serra Talhada, Pernambuco, Brazil.

Since there was no significant interaction effect, the factors (irrigation and biostimulant doses) were evaluated separately (Figs 6 and 7). Melon productivity and water use efficiency (WUE) were strongly influenced by irrigation depth and seasonal conditions, reflecting the interaction between water availability, crop phenology, and environmental constraints (Figs 6 and 7). Across all periods, yields were highest during the rainy season and declined markedly in the transition and dry periods, highlighting the sensitivity of melon growth to water stress and seasonal variations in climatic demand.

In the rainy season, productivity under 100% and 75% AW significantly surpassed that for the 50% AW treatment, demonstrating that sufficient irrigation is critical for maximizing fruit set and average fruit weight (Fig. 6). In contrast, during the transition and dry periods, productivity decreased across all irrigation levels, with reductions ranging from approximately 69% to 79% relative to the rainy season (Fig. 6). The greatest yield losses were consistently observed at 50% AW, reflecting the combined effects of limited water supply and increased evaporative demand.

WUE exhibited a complementary pattern across the cultivation periods. Although the highest absolute productivity occurred in the rainy season, WUE was lowest during this period due to the combined contribution of precipitation and irrigation (Fig. 7(B)). During the transition and dry periods, WUE increased, reaching peak values under limited irrigation. However, these high WUE values in low‐irrigation treatments were associated with markedly lower productivity, emphasizing that high WUE alone does not necessarily indicate optimal crop performance. This relationship underscores the importance of interpreting WUE in the context of yield rather than as an isolated metric (Fig. 7(B)).

The application of amino acid‐based biostimulants influenced productivity in a context‐dependent manner (Fig. 7). In the rainy season, moderate rates (4–6 L ha^−1^) tended to enhance productivity, with the maximum efficiency estimated at 4.95 L ha^−1^, corresponding to improved fruit development and higher average yields. During the transition and dry periods, biostimulant effects were less pronounced, and the highest rate (8 L ha^−1^) was associated with the largest reductions in productivity (Fig. 7), suggesting that under conditions of water stress, the potential of biostimulants to mitigate yield losses is limited. These observations indicate that biostimulant application is most beneficial when water availability is sufficient to support crop growth.

Overall, the results indicate that moderate biostimulant application rates (around 4–5 L ha^−1^) provided the highest yield (Fig. 6) and WUE (Fig. 7), while higher rates tended to reduce these variables.

Considering both irrigation and biostimulant treatments, the results indicate a clear hierarchy in melon performance: high irrigation levels (75–100% AW) consistently promoted superior yields, while lower irrigation (50% AW) constrained production, particularly under the transition and dry season conditions. Moderate biostimulant rates (4–6 L ha^−1^) can enhance productivity, but their effectiveness is limited when environmental constraints, particularly water deficit, are severe. The integration of productivity and WUE data reveals that maximum efficiency requires both sufficient water supply and strategic biostimulant application, emphasizing the importance of adaptive management according to seasonal conditions.

### Effect of salinity on productivity

**Figure 8:**
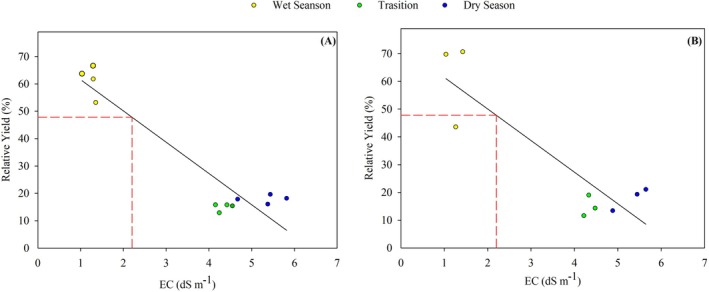
Relative productivity of melon as a function of ECe across three cultivation periods. (A) Effect of amino acid‐based biostimulant rates (0, 4, 6, 8 L ha^−1^). (B) Effect of irrigation levels (50, 75, 100% AW). Points highlighted in orange, blue, and green correspond to the rainy season, transition period, and dry season, respectively.

Soil salinity exerted a significant influence on melon productivity, particularly under conditions of limited water availability. Across the cultivation periods, relative productivity (Pr) was inversely related to ECe, indicating that higher salinity levels constrained fruit development and overall yield (Fig. 8).

At the end of the rainy season, plots irrigated at 75% and 100% AW exhibited ECe values of 1.03 and 1.42 dS m^−1^, respectively, corresponding to high relative productivity (Pr ≈ 70%) (Fig. 8). In contrast, the 50% AW treatment had an intermediate ECe of 1.26 dS m^−1^ and a lower Pr of 43.6%, below the critical threshold (ECe = 2.2 dS m^−1^; Pr = 47.8%) estimated from linear regression. These results indicate that under adequate irrigation, soil salinity remained below levels that would substantially limit yield, allowing plants to achieve high productivity (Fig. 8).

During the transition and dry periods, soil salinity increased considerably, with ECe values exceeding 4.0 dS m^−1^ across treatments (Fig. 8). Under these conditions, Pr declined sharply (Pr < 20%), regardless of biostimulant application or irrigation level. This trend highlights that high salinity acts as a dominant stress factor, overwhelming the potential benefits of biostimulants and limiting the effectiveness of irrigation management under water‐limited conditions.

Biostimulant application had a measurable effect on productivity only when salinity was below critical thresholds. For instance, in the rainy season, 4 L ha^−1^ of biostimulant improved Pr to 66.6% under an ECe of 1.52 dS m^−1^ (Fig. 8), demonstrating that amino acid‐based stimulants can enhance melon performance under low‐stress conditions. However, as salinity increased during the transition and dry periods, biostimulant effects were minimal, suggesting that environmental stress, rather than crop management, was the primary limitation on productivity in these periods.

These results emphasize the interdependence between irrigation, soil salinity, and productivity. Effective melon cultivation requires maintaining soil salinity below critical levels, particularly in periods of water deficit, to maximize yield potential. Biostimulants can complement irrigation management, but their efficacy is contingent on favorable environmental conditions, highlighting the importance of integrated water and salinity management strategies in melon production.

## DISCUSSION

Although the interaction between irrigation levels and biostimulant application was not statistically significant (*P* > 0.05), the results indicate that the response to the biostimulant occurred independently of irrigation level. This suggests that the beneficial effects of the biostimulant were expressed across all irrigation conditions rather than being restricted to a specific water deficit level. Biostimulants are known to enhance plant physiological processes such as nutrient uptake, photosynthetic efficiency, and tolerance to abiotic stresses,^32^ which may contribute to improved plant performance even under deficit irrigation. Therefore, the application of biostimulants may represent a complementary strategy to improve crop resilience and WUE in semi‐arid environments.^33^

The reduction in temperature and solar radiation had adverse impacts, especially in the initial phase of the crop, compromising water absorption and photosynthesis.^34^ Throughout the three cultivation periods, considerable changes were observed in the variables of temperature, ET_0_, and radiation, with the transition period recording the lowest values for these three variables compared to the others. The main melon crop, which occurs between February and April, is characterized by optimal thermal conditions for plant development, with ideal temperatures ranging from 27 to 33 °C. The average relative humidity during the cultivation periods was 64.77% in the rainy season, 60.69% in the transition period, and 46.82% in the dry season.^7^ In the rainy season and transition period, the emergence of diseases such as that caused by *Didymella bryoniae* was observed, resulting in the mortality of some plants. Temperatures between 20 and 26 °C, combined with high relative humidity, favored the development of the fungus.^35^ Successive cultivation may have contributed to the increase in the pathogen's inoculum source through possible crop residues; however, in the dry season, no plant mortality due to the fungus was observed. It is important to note that melon cultivation without irrigation is unfeasible, as between 300 and 550 mm of water per cultivation period are required, depending on the local climatic conditions of the region.^7^

Melon shows moderate tolerance to salinity, with a critical threshold around 2.2 dS m^−1^ in the saturation extract; however, when salinity levels exceed this limit, detrimental effects on both yield and fruit quality become evident.^8^ This is evidenced in our work, where in the rainy season, ECe is lower than the critical threshold, and productivity is higher compared to the transition and dry seasons, where ECe was above 4.0 dS m^−1^. Maintaining the electrical conductivity of the soil saturation extract below 1.2 dS m^−1^ is crucial to ensure optimal cultivation conditions. The literature highlights that, upon reaching a conductivity level of 1.8 dS m^−1^, a yield reduction of around 10% is expected. This impact increases significantly, reaching a predicted reduction of 50% when conductivity reaches 4.3 dS m^−1^, and an expected reduction of 100% at 7.5 dS m^−1^.^37^ The increase in soil electrical conductivity had an adverse effect on the growth and development of melon plants, resulting in a significant reduction in fruit production and size. This phenomenon is attributed to the restriction in water absorption caused by the accumulation of toxic ions, such as Na^+^ and Cl^−^, as highlighted by Ahmed *et al*.^36^

These results corroborate those found in studies showing that under adequate water supply stomatal conductance typically reaches its highest values around solar noon, and in our work most afternoon measurements also presented increased stomatal conductance. Stomatal conductance can limit water loss and regulates gas exchange between the plant and its environment, playing an important role in mitigating water stress (e.g. recent findings on the relationship between stomatal conductance and photosynthesis under water stress conditions in crop species).^37^ Our data corroborate findings that water stress influences physiological responses in melon, particularly in stomatal conductance and gas exchange mechanisms that help regulate water loss under deficit conditions. This is consistent with results showing how varying irrigation levels affect these physiological parameters in drought‐tolerant melon genotypes. Stomata are responsible for controlling gas exchanges and regulating water loss in plants, helping reduce water stress.^33^

These findings reinforce the importance of proper management of biostimulants to improve plant resistance to water stress, enhancing the sustainability of agricultural production in semi‐arid regions. This is attributed to the conditions of low water content in the soil, which, by reducing cell turgor, decreases stomatal conductance and transpiration, increasing leaf temperature and its amplitude throughout the day.^38^ Although no significant interaction between irrigation levels and biostimulant application was observed, the combined analysis of these factors is important for understanding plant responses under water‐limited conditions. In general, deficit irrigation tends to reduce plant growth and productivity due to limitations in water uptake and photosynthetic activity. However, the use of biostimulants may help alleviate some of these effects by stimulating physiological processes associated with stress tolerance, such as improved nutrient absorption, osmotic adjustment, and antioxidant activity. In this context, the use of biostimulants together with deficit irrigation strategies may contribute to maintaining plant performance under reduced water availability, representing a promising management strategy for semi‐arid agricultural systems.^18^ Akhoundnejad and Dasgan,^33^ working with water stress in melon crops in different genotypes, found leaf temperature values ranging from 26.25 to 33.03 °C, which are lower than those found in our research. The absence of water and the presence of salinity cause stomatal closure, reducing the transpiration rate and causing an increase in leaf temperature in the canopy.^5^

The levels of chlorophyll a, chlorophyll b, and carotenoids in melon crops (Table 7) did not show significant differences between treatments within each studied period. However, it is possible to observe that the levels of chlorophyll a and chlorophyll b in the rainy and transition seasons are higher than those found in the dry season. This decline in chlorophyll levels over the periods can be attributed to saline stress, which results in the presence of toxic ions, dehydration of mesophyll cells, and inhibition of various enzymes involved in carbohydrate metabolism and chlorophyll synthesis.^32^ Since chlorophyll is the primary pigment for light energy absorption, its reduction leads to a decrease in the photosynthesis rate. Therefore, measuring chlorophyll content can be used as an indicator of plant stress.^36^ The negative influence of salinity on chlorophyll content in melon has been widely reported in previous studies, corroborating the results observed in the present study.^11^ It is also observed that, despite the absence of significant differences between treatments, carotenoid levels also showed variations between different periods, being generally lower in the dry season, which may be related to the conditions of water and saline stress that affect the synthesis and stability of these pigments.

The number of fruits per plant is one of the crucial factors in determining the productivity of melon crops. Studies evaluating different irrigation system arrangements and soil cover in melon cultivation reported fruit number values of approximately 1.75 fruits per plant for the same hybrid used in this study, while experiments assessing water stress at different phenological stages of Piel de Sapo melon observed fruit numbers ranging from 2.3 to 2.6 fruits per plant.^39^ Studies evaluating melon cultivation under water stress reported equatorial diameter values of 11.65 cm for yellow melon, which are lower than those observed in the present study, whereas equatorial diameter values ranging from 15.33 to 16 cm have been reported under different management conditions, corroborating our results. Longitudinal diameter values between 14.98 and 17.93 cm have also been described, similar to those obtained during the rainy season in this study. Variations in fruit length may be associated with differences in sink strength during the cell division phase as well as with fruit growth rates during the cell expansion phase. In addition, rind firmness values for yellow melon have been reported to range from 17.91 to 45.01 N, depending on cultivation conditions and management practices.^7^, ^10^, ^35^

For the transition period and dry season, no significant difference was observed for irrigation levels. However, when comparing crops in different periods, it is evident that there was a reduction in fruit pulp firmness in the transition period compared to the rainy and dry seasons. The reduction in rind and pulp firmness of the fruits is directly related to the low absorption of nutrients, especially calcium, due to the presence of high salinity levels in the soil. The high concentration of salts interferes with the absorption of Ca^2+^ by the plants, reducing water availability and consequently decreasing fruit pulp firmness. The Ca^2+^ ion plays a fundamental role in the formation and stability of cell walls, acting in the middle lamella and contributing to cell rigidity.^8^

The transition period showed an average reduction in fruit pulp firmness for irrigation levels and biostimulant rate by 5.88 and 6.57 N compared to the rainy and dry seasons. It is important to highlight that the reduction in water availability can impact the development of the fruit pulp, affecting its firmness, an essential characteristic for fruit quality and shelf‐life extension.^17^ Current literature indicates that fruit quality parameters such as flesh firmness, total soluble solids, and TA in melon are influenced by irrigation management and water availability, with water deficit often modifying these traits without always impairing marketable quality. In melon fruits, soluble solids and TA may remain stable across different irrigation conditions, while firmness and sugar accumulation can vary with plant water status and phenological stage, affecting quality attributes relevant for postharvest performance and consumer acceptance.^38^, ^40^, ^41^

The increase in soil electrical conductivity due to salt accumulation negatively affected crop growth and development, resulting in a significant reduction in fruit production. Soil salinity has emerged as a significant concern, as high levels of salts can harm crops. Melon is moderately sensitive to salinity, and critical salinity levels have been identified. During the transition period and dry season, the study revealed a significant reduction in almost all postharvest variables due to the influence of salinity and climatic conditions, highlighting the importance of monitoring and managing soil water and salinity. The drop in productivity in the dry season compared to the rainy season is also evident, with reductions for the 50%, 75%, and 100% AW levels of 74.70%, 79.47%, and 73.19%, respectively, surpassing the reduction in the transition period. Previous studies have reported productivity values ranging from 24.46 to 32.15 Mg ha^−1^, as well as yields of around 34.41 Mg ha^−1^, which are comparable to those observed during the rainy season in the present study. In contrast, investigations evaluating different irrigation systems under saline conditions have reported lower productivity values, ranging from 19.05 to 26.00 Mg ha^−1^, highlighting the negative effects of salinity on melon yield.^41^, ^42^ This response suggests that irrigation management plays a major role in determining melon productivity, while the application of biostimulants may contribute to improving plant tolerance to environmental stresses without necessarily altering the response pattern among irrigation levels.

Plants irrigated with 50, 75, and 100% AW in the rainy season showed WUE of 0.049 ± 0.016, 0.075 ± 0.011, and 0.068 ± 0.016 Mg mm^−1^ ha^−1^, respectively (Fig. 3(B)). In the transition period, there was also a significant difference between the levels, with WUE of 0.120 ± 0.086, 0.143 ± 0.091, and 0.125 ± 0.043 Mg mm^−1^ ha^−1^ for the same levels. In the dry season, the WUE found for the studied levels was 0.308 ± 0.025, 0.195 ± 0.055, and 0.308 ± 0.032 Mg mm^−1^ ha^−1^. It is observed that the highest WUE was in the dry season for the 50% AW level. However, this does not necessarily translate into something positive due to the low productivity found for the same level in this period. Under stress conditions, plants tend to reduce stomatal conductance and transpiration as a strategy to optimize WUE, which is consistent with the results observed in this study during cultivation in the dry season. In addition, the increase in soil salinity reduces the osmotic potential, thereby compromising water absorption by plants, which may explain the lower water consumption observed during the last two cultivation periods.^5^, ^20^

## CONCLUSIONS

Melon productivity and WUE were strongly influenced by irrigation level and cultivation period. High irrigation levels (75–100% AW) consistently promoted higher yields across all periods, particularly during the rainy season, whereas reduced irrigation (50% AW) resulted in significant declines, especially during the transition and dry seasons. Soil salinity emerged as a major limiting factor, with electrical conductivity exceeding 4 dS m^−1^ during the transition and dry periods, leading to relative productivity below 20%. These results indicate that water availability and salinity interact to determine melon performance, often outweighing the effects of biostimulant application under stressful conditions. Moderate rates of amino acid‐based biostimulants (4–6 L ha^−1^) improved yields primarily under low salinity and moderate water stress, suggesting that their effectiveness is contingent on favorable environmental conditions.

Taken together, the findings highlight the importance of integrated crop management that combines optimized irrigation levels, salinity monitoring, and judicious biostimulant use. Maintaining adequate water supply and mitigating salt accumulation in the root zone are essential for sustaining productivity and achieving efficient water use. These results provide a framework for enhancing melon yield under varying climatic conditions while supporting soil health and long‐term sustainability. Future research could further explore adaptive irrigation strategies and biostimulant applications to maximize crop performance under saline and water‐limited environments.

## CONFLICT OF INTEREST

The authors declare that they are not aware of any financial or personal conflicts of interest that could have influenced the work reported in this article.

## FUNDING INFORMATION

This research was supported by CAPES – Coordination for the Improvement of Higher Education Personnel (process nos. 88887.136369/2017‐2100 [INCT – OndaCBC] and 88887.686398/2022‐2100); FACEPE – Foundation for Science and Technology Support of the State of Pernambuco (APQ‐0498‐3.07/17 [INCT – OndaCBC], APQ‐1737‐5.01/22, and APQ‐0238‐3.14/23); and CNPq (process no. 465764/2014‐2 [INCT – OndaCBC]; UFRPE – Federal Rural University of Pernambuco and CNPq – National Council for Scientific and Technological Development).

## Data Availability

Research data are not shared.
